# Tibial loading and damage accumulation in recreational and competitive male runners during a demanding 10 km run

**DOI:** 10.1002/ejsc.12040

**Published:** 2024-01-30

**Authors:** Hannah Rice, Patrick Mai, Maximilian Sanno, Steffen Willwacher

**Affiliations:** ^1^ Department of Physical Performance Norwegian School of Sport Sciences Oslo Norway; ^2^ Institute for Advanced Biomechanics and Motion Studies Offenburg University Offenburg Germany; ^3^ Institute of Biomechanics and Orthopaedics German Sport University Cologne Cologne Germany

**Keywords:** cumulative loading, musculoskeletal modeling, overuse injury, tibial stress

## Abstract

Tibial stress injuries are problematic among runners. The loading magnitude is the most important mechanical contributor to bone damage accumulation, but loading quantity is also important, and faster runners require fewer loading cycles to complete a given distance than slower runners. This study estimated tibial loading and damage accumulation throughout a demanding 10‐km run in recreational (RR) and competitive runners (CR). Male runners reporting a 10‐km season‐best run slower than 47:30 min (RR) or faster than 37:30 min (CR) completed a 10‐km treadmill running protocol at 105% of their season's best time. Tibial loading was estimated from bending moments at the distal 1/3^rd^ of the tibia by ensuring static equilibrium at each 1% of stance. Peak loading was obtained, and cumulative damage per kilometer was estimated using a tissue‐dependent weighting factor. Peak tibial loading and damage accumulation per kilometer significantly decreased throughout the run, by 5% and 4%, respectively. Peak loading was significantly higher (31%) in CR than RR, and there was an indication (*p* = 0.058 and large effect size) of a greater rate of damage accumulation in CR than RR. Tibial loading per step and the rate of accumulation per kilometer decreased throughout a demanding 10‐km run suggesting that changes in running mechanics as a result of prolonged running may not be a primary mechanism for stress injury development. Competitive runners experience greater peak tibial loading and possibly greater cumulative tibial damage when they complete 10 km faster than recreational runners.

## Highlights

- Peak tibial loading per step and the rate of damage accumulation decreased throughout a demanding 10‐km run in both recreational and competitive runners.
- Running‐induced changes in gait mechanics may not be a predominant mechanism for the development of tibial stress injuries.
- Completing a 10‐km run at a faster speed requires fewer loading cycles, but this does not result in reduced tibial damage accumulation due to the greater loading magnitude.

## 1 INTRODUCTION

Runners are at relatively high risk of lower limb bony stress injuries due to the repetitive nature of running, which leads to the accumulation of microdamage in the bone (Burr et al., 1998). The magnitude of loading experienced by the bones is considered to contribute most to the risk of stress fracture (Carter et al., 1981; Edwards, 2018; Loundagin et al., 2018) while the quantity (i.e., the duration and number of loading cycles) is also influential (Burr, 1997; Edwards, 2018; Firminger et al., 2016). Conversely, the rate of loading is not deemed to be related to increased risk of bone injury from a mechanical perspective (Edwards, 2018; Loundagin et al., 2018).

Increasing running speed increases the magnitude of tibial loading per step (Edwards et al., 2010; Meardon et al., 2021) but also alters the quantity of loading. Increased running speed can lead to both reduced contact time (Brughelli et al., 2011; Cavanagh et al., 1989; Clark et al., 2014) and increased stride length (Weyand et al., 2000), which in turn results in a reduction in the number of steps per given distance. As such, the combined influence of altered magnitude and quantity of loading is important to consider in the context of risk of stress injury. Measures of cumulative loading account for both the magnitude and quantity of loading. The relative importance of magnitude should be accounted for to avoid placing equal weighting on the magnitude and quantity of loading (Edwards, 2018). This can be achieved using a ‘weighted impulse’, as previously proposed (Edwards, 2018; Firminger et al., 2020), by raising the loading magnitude to a power that is specific to the tissue of interest. This provides an indirect estimate of cumulative ‘damage’, which is very difficult to directly measure in vivo.

Changes to the loading and damage accumulation experienced by the tibia through the course of a prolonged run are not well understood. Increased tibial loading magnitude as a result of running‐ and/or marching‐induced fatigue has previously been suggested to be a mechanism for stress fracture development (Milgrom et al., 2007). However, it is unclear whether completing a run alters loading magnitude, as existing findings have been contradictory. Increased strain, measured using a strain gauge (Milgrom et al., 2007), and increased anterior and posterior tibial stress, estimated using beam theory modeling (Rice et al., 2019) have been reported after a 2‐km run and 20 min of treadmill running, respectively. Conversely, Khassetarash et al. 2023 reported no change in peak tibial strain or strained volume after 30 min of downhill running, while Burnett (2017) reported reduced peak strain after running for ∼113 min. In terms of quantity of loading, runners reportedly adapt their running gait throughout a run, even when velocity is held constant (Hanley and Mohan, 2014). Hanley and Mohan (2014) found that over the course of a 10‐km treadmill run at a constant velocity, there was a decrease in both contact time and step frequency. The combination of altered magnitude and quantity of loading throughout a treadmill run may have implications for bone loading and risk of injury. In a real‐world setting, competitive runners are able to complete a given distance at a faster speed than recreational runners. This will likely result in a higher magnitude of tibial loading but a reduced quantity, such that the accumulation of tibial damage may not be so different between the groups, and this warrants quantification. It is important to quantify changes in loading throughout the duration of a run, as this may help to understand mechanisms for the development of tibial stress injuries. Furthermore, there may be a difference in the extent to which changes in tibial loading during a run occur between runners of different levels, as a result of their adaptations to training. Any differences may provide an indication as to whether level of running can be protective against stress injury development.

The aim of this study was to estimate internal loading of the tibia at the distal 1/3^rd^ centroid during a 10‐km treadmill run, and to compare both peak and cumulative loading in recreational and competitive runners while running at 105% of their individual season's best 10‐km time. It was hypothesized that (1) peak tibial loading per step and damage accumulation per kilometer would change throughout the duration of the run; (2) competitive runners would demonstrate greater peak tibial loading than recreational runners while running at a faster running speed; (3) competitive runners would demonstrate greater cumulative loading throughout 10 km than recreational runners, despite requiring fewer steps, due to the greater importance of magnitude than quantity of loading.

## 2 MATERIALS AND METHODS

### 2.1 Participants

Two groups of runners (*n* = 24), categorized as recreational and competitive, participated in this study, which was a further analysis of a previously acquired dataset (Sanno et al., 2018, 2021; Willwacher et al., 2020). Participants were injury free in the 12 months prior to data collection, had run regularly for at least 2 years, and had experience of treadmill running. Participants categorized as recreational runners (RR) included students (*n* = 13; age 24.3 ± 3.4 years; height 1.84 ± 0.05 m; and mass 81.3 ± 7.4 kg) with a self‐reported season‐best slower than 47:30 min in a 10‐km run. The group of competitive runners (CR) included distance runners (*n* = 11; age 24.7 ± 3.8 years; height 1.82 ± 0.06 m; and mass 73.0 ± 7.9 kg) with a self‐reported season‐best faster than 37:30 min in a 10 km run. A power calculation in G*Power 3.1.9.7 (Faul et al., 2007) suggested this sample size was suitable based on previous values of peak bending moments at the tibia when running at slower and faster speeds (Rice et al., 2023), with a power of 0.8. Participants signed written informed consent before taking part. The local research ethics committee of the German Sport University Cologne approved the study protocol.

### 2.2 Experimental protocol

Following a warm‐up at a self‐selected running speed for at least five minutes, participants performed a 10‐km treadmill run at 105% of their season‐best time (RR: 52:49 ± 2:21 min; CR: 37:32 ± 1:17 min) on a single‐belt treadmill (Treadmetrix, Park City, USA). The treadmill was equipped with four 3D force transducers (1000 Hz, bMC3A‐3‐500‐4876; AMTI Inc., Watertown, USA) to collect ground reaction forces. A marker‐based motion capture system (250 Hz, 13 MX‐F40 cameras; Vicon Motion Systems, Oxford, UK) captured the position of 78 retroreflective markers (diameter: 13 mm) attached to the runner's lower and upper body. Skin‐friendly glue (Mastix, Kultfaktor GmbH, Metzingen, Germany) and double‐sided adhesive Toupee Tape (Kryolan, Berlin, Germany) were used to prevent markers from falling off. Markers at the foot were attached to the shoe's surface. A detailed description of the procedure can be found in previous publications (Sanno et al., 2018, 2021; Willwacher et al., 2020). All participants wore the same racing flat shoes (Adizero Pro 4; Adidas AG). A familiarization run was performed 7 days before the data acquisition to allow participants' footwear and treadmill customization. Data were collected at 0 km and each kilometer completed thereafter until 10 km.

### 2.3 Data analysis

Marker trajectories and ground reaction force data were filtered with a recursive, fourth‐order digital Butterworth filter with a cutoff frequency of 20 Hz (Mai et al., 2019). A three‐dimensional inverse dynamics model, consisting of 15 rigid body segments, was used to calculate 3D joint angles and external joint moments at the hip, knee, and ankle joints (Sanno et al., 2018). The joint angles were referenced to the static posture obtained during a standing reference trial.

The bending moments about the medial–lateral (ML) tibial centroid axis (i.e., sagittal plane) contribute to anterior and posterior tibial stresses. ML bending moments provide an indication of tibial loading and were estimated at a centroid representing the distal one‐third of the tibia in MATLAB (R2021a, MathWorks) in a customized program, using methods similar to those reported previously (Baggaley et al., 2021; Meardon et al., 2014; Rice et al., 2019). Internal forces and moments were estimated by ensuring static equilibrium at each 1% of the stance phase (Baggaley et al., 2021; Rice et al., 2019). Muscular forces were estimated from 11 muscles that span the tibial centroid. The muscular force lines of action were determined as previously (Rice et al., 2019), based on open source musculoskeletal models (Delp et al., 1990; Hamner et al., 2010). Muscular forces were estimated using a customized MATLAB static optimization program with a cost function minimizing the sum of cubed muscle stresses (Rice et al., 2019). The program was constrained so that the muscular forces were equal to the hip, knee, and ankle joint moments in the sagittal plane (Rice et al., 2019). The resultant ML bending moment was the sum of the moments due to joint reaction forces and muscular forces.

Step frequency was averaged for each kilometer completed, and the number of steps required to complete 1 km for the participant's given speed and step frequency was obtained. This allowed estimation of cumulative damage using a weighted impulse, as in Equation (1) proposed by Firminger et al. (2020):

(1) $$\text{Cumulative}\;\text{damage}={\left[n\int_{t_{i}}^{t_{f}}{\left(x_{s}\right)}^{b}\;dt\right]}^{1/b}$$

where *n* is the number of right foot contacts, *t*_i_ = beginning of stance, *t*_f_ = end of stance, x_s_ is the measure of internal bone loading (i.e., bending moment time series), and *b* is the tissue‐dependent weighting factor that represents the power function between fatigue life and applied stress/strain from experimental data (Firminger et al., 2016). The constant *b* here for bone is equal to 6.6 (Carter et al., 1985). Using a weighted impulse accounts for the fact that the magnitude of loading is more important than the number of loading cycles in terms of bone damage accumulation (Edwards, 2018; Firminger et al., 2020).

Ten steps per participant were analyzed from kilometer 0 and after each kilometer completed throughout the 10‐km run and averaged per participant. Peak ML bending moments were extracted. Cumulative damage was estimated as in Equation (1) for each kilometer of the run from the ML bending moment time series by calculating the impulse with respect to time. The number of steps was derived from the average step frequency for each kilometer completed and divided by two to represent damage accumulated by the right tibia only. The damage accumulated per kilometer was estimated and the sum per kilometer completed was reported (rate of accumulation). Ground contact time (GCT), step frequency, and step length normalized to height were also reported to aid explanation of the main findings.

### 2.4 Statistical analysis

Statistical analyses were conducted in SPSS (IBM SPSS Statistics for Windows, Version 28.0, IBM Corp) and MATLAB (R2021a, MathWorks) with a significance level of 0.05. A two‐way mixed ANOVA with Distance Completed (11 time points for temporal and peak variables; 10 time points for cumulative damage) as within‐subjects factor and Running Level (2 groups) as between‐subjects factor was conducted. Where Mauchly's test of sphericity was significant, the Greenhouse–Geisser correction was used. Partial eta squared (*η*_p_^2^) indicated effect size for the main effects and were interpreted as small (0.01–0.059), medium (0.06–0.139), and large (0.14) (Cohen, 1988). Cohen's D effect sizes were reported (Cohen, 1988) and interpreted as trivial (*d* = 0–0.19), small (*d* = 0.2–0.49) medium (*d* = 0.50–0.79), or large (*d* ≥ 0.8).

## 3 RESULTS

### 3.1 Peak bending moments

**FIGURE 1:**
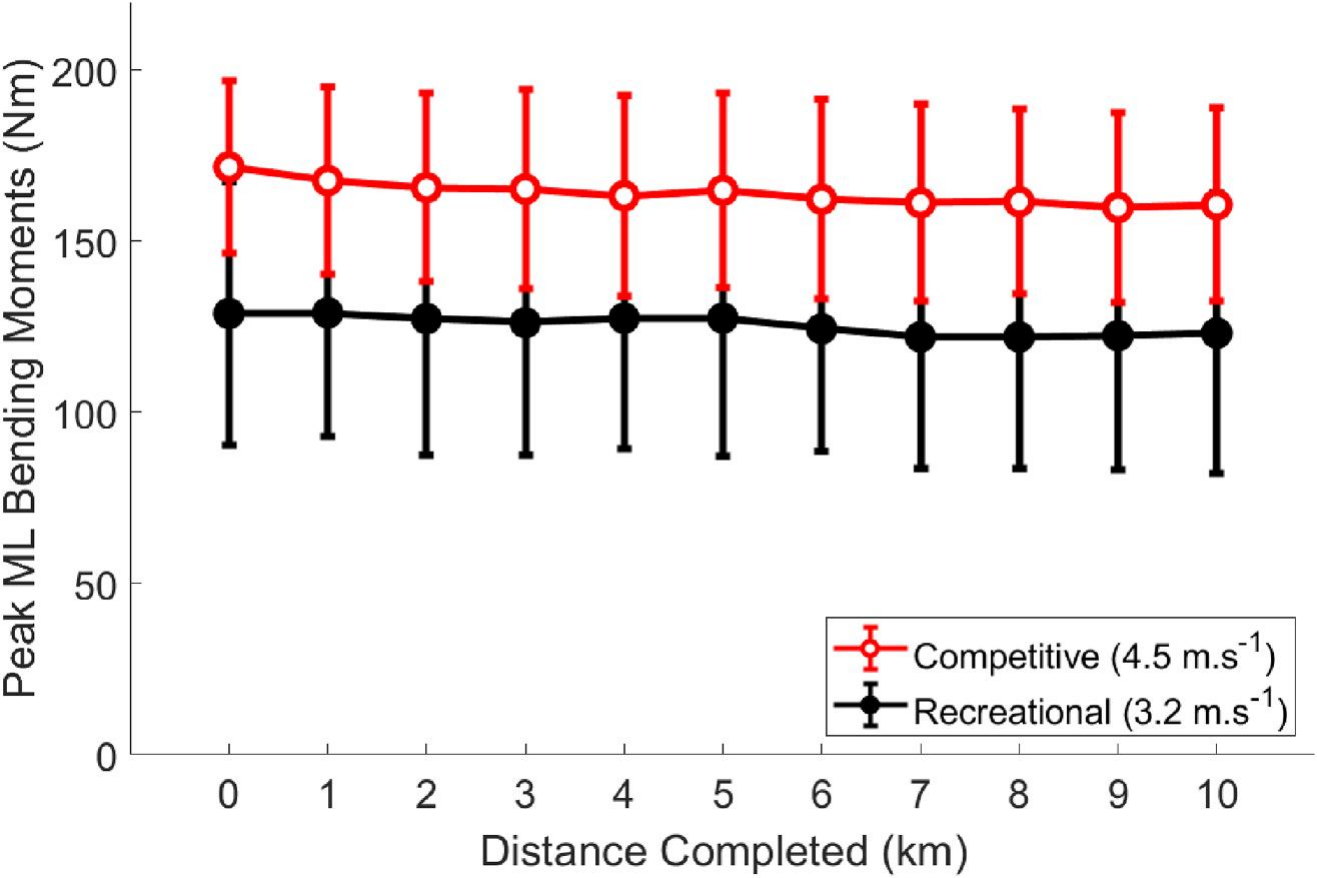
Mean (SD) peak ML bending moments about the distal third of the tibia for each kilometer of running in RR and CR. Peak bending moment values were negative, indicating posterior compression and anterior tension. Absolute values are presented here.

Peak ML bending moments were in the negative direction, indicative of posterior compression and anterior tension. There was no interaction effect (*F*_(3.223)_ = 0.526 and *p* = 0.679) for peak bending moments (Figure 1); thus, main effects were considered. There was a main effect for Distance Completed (*F*_(3.223)_ = 6.022 and *p* < 0.001, *η*_p_^2^ = 0.215), whereby peak ML bending moments decreased throughout the run by 5% on average from the start to the end of the run. There was a main effect for Level of Running (F_(1)_ = 7.753, *p* = 0.011, and *η*_p_^2^ = 0.261), whereby peak ML bending moments were 31% higher in the competitive runners than the recreational runners on average throughout the run.

### 3.2 Rate of damage accumulation

**FIGURE 2:**
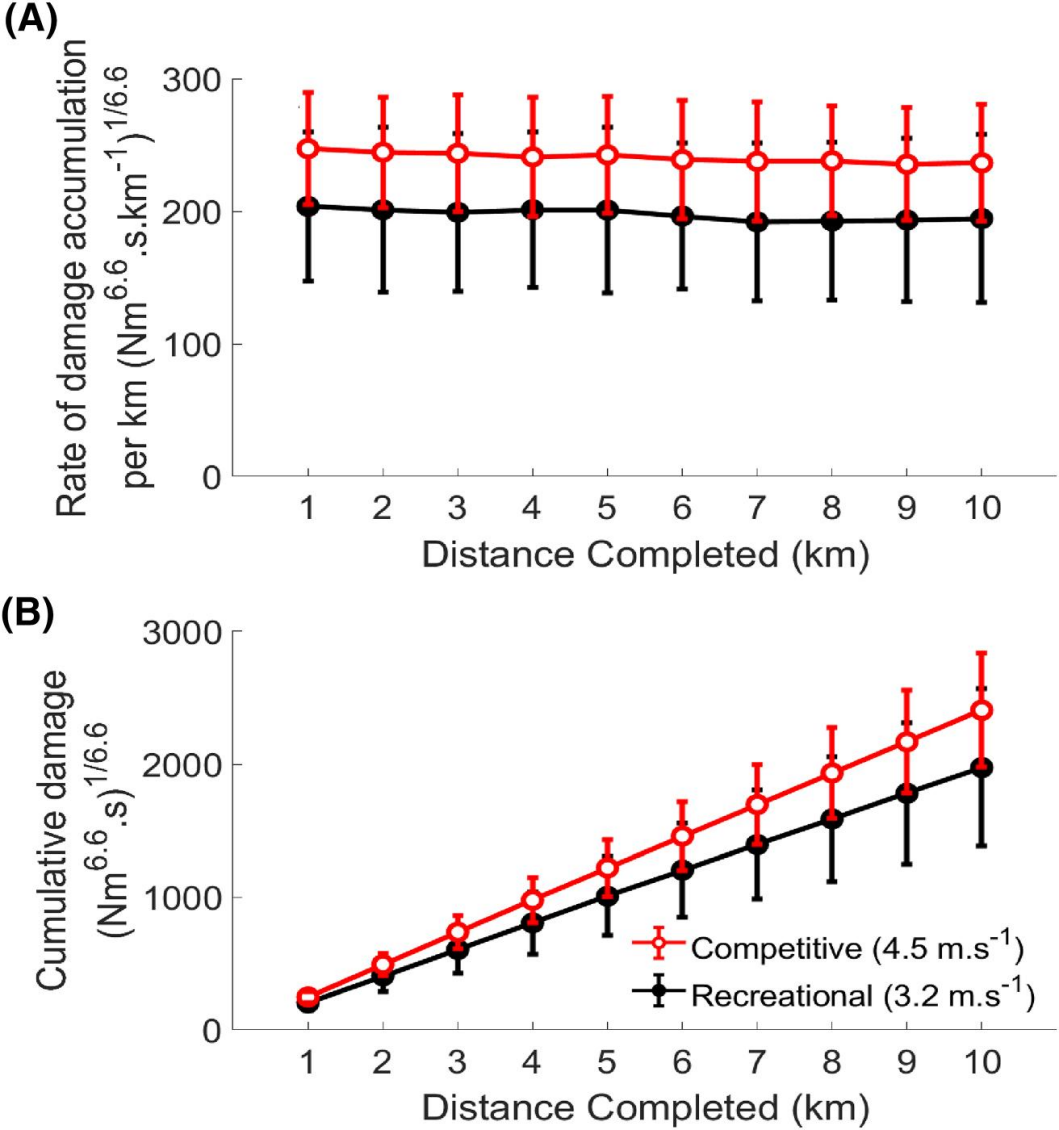
(A). Mean (SD) rate of damage accumulation per kilometer at the distal third of the tibia for each kilometer of running in RR and CR. (B): Mean (SD) cumulative damage at the distal third of the tibia for each kilometer of running in RR and CR. Cumulative damage is the sum of the rates of accumulation per kilometer for each kilometer completed.

There was no interaction effect (*F*_(3.435)_ = 0.283 and *p* = 0.863) on the rate of damage accumulation per kilometer (Figure 2). There was a main effect for Distance Completed (*F*_(3.435)_ = 5.316, *p* = 0.001, and *η*_p_^2^ = 0.195), whereby the rate of damage accumulation per kilometer decreased throughout the run by 4% on average from the start of the run to the end (Figure 2A). There was no main effect for running level (F_(1)_ = 4.013, *p* = 0.058, and *η*_p_^2^ = 0.154), but a *p* value < 0.1 and large effect size indicated greater damage accumulation per kilometer in the competitive runners than recreational (Figure 2B). Competitive runners accumulated 22% greater tibial damage than recreational runners at the end of the 10 km run on average, if the difference between the groups can be considered meaningful based on the large effect size.

### 3.3 Spatial‐temporal variables

Mean (SD) running speed was 3.16 (0.14) m/s for recreational runners and 4.45 (0.16) m/s for competitive runners. No statistically significant interaction effect was observed for GCT (*F*_(3.490)_ = 0.450 and *p* = 0.747). However, there were statistically significant main effects for Distance Completed (*F*_(3.490)_ = 4.950, *p* = 0.002, and *η*_p_^2^ = 0.184) and Running Level (*F*_(1)_ = 45.291, *p* < 0.001, and *η*_p_^2^ = 0.673) (Table 1). On average, the GCT for competitive runners was 51 ms shorter than the GCT for recreational runners. GCT steadily increased from the start of the run and was longest after 8 km. This occurred regardless of the Running Level.

**TABLE 1:** Mean (SD) ground contact time, step frequency per kilometer, and step length normalized to height for recreational runners (RR) and competitive runners (CR), including statistical outcomes from the two‐way mixed ANOVA.

		Running kilometer													
		0	1	2	3	4	5	6	7	8	9	10	Running level	Distance completed	Group x distance
Ground contact time [ms]	**RR**	252	252	257	255	256	256	257	257	258	257	255	*p* < 0.001	*p* = 0.002	*p* = 0.747
		(21)	(21)	(22)	(23)	(23)	(24)	(25)	(23)	(23)	(24)	(23)			
	**CR**	202	203	205	205	206	205	206	206	207	206	204	*η*_p_^2^ = 0.673	*η*_p_^2^ = 0.184	
		(12)	(12)	(11)	(12)	(11)	(11)	(11)	(14)	(14)	(13)	(13)			
Step frequency [Hz]	**RR**	2.5	2.5	2.5	2.5	2.5	2.5	2.5	2.5	2.5	2.5	2.5	*p* = 0.017	*p* = 0.062	*p* = 0.805
		(0.1)	(0.1)	(0.1)	(0.1)	(0.1)	(0.1)	(0.1)	(0.1)	(0.1)	(0.1)	(0.1)			
	**CR**	2.7	2.7	2.7	2.7	2.7	2.7	2.7	2.7	2.7	2.7	2.6	*η*_p_^2^ = 0.234		
		(0.2)	(0.2)	(0.1)	(0.1)	(0.1)	(0.2)	(0.2)	(0.2)	(0.2)	(0.2)	(0.2)			
Normalized step length	**RR**	0.68	0.68	0.69	0.68	0.68	0.68	0.68	0.69	0.68	0.68	0.69	*p* < 0.001	*p* = 0.047	*p* = 0.723
		(0.04)	(0.04)	(0.04)	(0.04)	(0.04)	(0.04)	(0.04)	(0.04)	(0.04)	(0.04)	(0.05)			
	**CR**	0.88	0.89	0.89	0.88	0.88	0.89	0.89	0.89	0.88	0.89	0.89	*η*_p_^2^ = 0.806	*η*_p_^2^ = 0.095	
		(0.04)	(0.04)	(0.04)	(0.04)	(0.04)	(0.04)	(0.04)	(0.04)	(0.04)	(0.04)	(0.04)			

There was no interaction effect (*F*_(5.203)_ = 0.470 and *p* = 0.805) or main effect of Distance Completed (*F*_(5.203)_ = 2.148 and *p* = 0.062) on step frequency (Table 1). There was a main effect of Running Level on step frequency (*F*_(1)_ = 6.727 and *p* = 0.017, *η*_p_^2^ = 0.234). On average, competitive runners ran with a step frequency that was 0.15 Hz faster than recreational runners.

There was no interaction effect on step length (*F*_(5.168)_ = 0.577 and *p* = 0.723) (Table 1). There was a main effect of Distance Completed for step length (*F*_(5.168)_ = 2.303 and *p* = 0.047), whereby normalized step length increased by 1.8% on average from the start to the end of the run. There was a main effect of Running Level on step length (*F*_(1)_ = 91.238, *p* < 0.001, and *η*_p_^2^ = 0.806). Competitive runners ran with a step length that was 30% longer than recreational runners when normalized to height and 34% longer in absolute terms (CR: 1.67 (0.12) m versus RR: 1.25 (0.07) m).

## 4 DISCUSSION

This study estimated internal loading of the tibia throughout a 10‐km run in both recreational and competitive runners, at 105% of their season‐best time. The direction of the ML bending moment indicated that the anterior tibia was predominantly under tension throughout stance, whilst the posterior tibia was predominantly under compression, in line with earlier findings (Baggaley et al., 2021; Derrick et al., 2016; Meardon et al., 2014, 2015; Rice et al., 2019, 2023; Yang et al., 2014). As hypothesized, the competitive runners experienced greater peak tibial bending moments than the recreational runners. This was assumed to be mostly influenced by the faster running speeds of the competitive runners. Competitive runners ran 41% faster than recreational runners on average, and peak tibial loading was 31% higher. The magnitude of the peak loading is more important than the quantity of loading (Edwards, 2018), but the quantity of loading (both in terms of ground contact time and the number of steps per kilometer) is reduced while running at a faster speed. Therefore, cumulative damage was considered, and there was evidence (*p* < 0.1 and large effect size) of a higher rate of damage accumulation and total cumulative load over 10 km in competitive than recreational runners. This suggests that completing a given running distance at a faster speed could increase the risk of bony stress injury development per 10 km, and supports previous suggestions that running at slower speeds reduces the risk of stress injury (Edwards et al., 2010). The large effect size but 0.05 < *p* < 0.1 suggests that the study was underpowered for this specific variable.

There was a decrease in both the peak tibial loading per step and the rate of cumulative damage per kilometer throughout the run, which confirmed the hypothesis. Peak tibial loading decreased from the start to the end of the run in 19 out of 24 participants (79%), where 69% of recreational runners and 91% of competitive runners experienced a decrease. Furthermore, the average decrease in peak ML bending moment from the start to the end of the run was 4.6% and 6.9% in recreational and competitive runners, respectively. This is likely due predominantly to the respective 3.0% and 4.8% decreased in bending due to muscular forces observed between the start and the end of the run. This does not support previous findings which showed increased in vivo tensile strain following a 2‐km run (Milgrom et al., 2007) and increased tibial loading following a 20 min treadmill run (Rice et al., 2019). The reduction in peak loading occurred after kilometer seven of the 10 km run on average in the present study. The methods used by Milgrom et al. (2007) were different from those in the present study; they quantified tibial strain using surgically implanted strain gauges in four participants and found a 35% increase in tensile strain. Rice et al. (2019) reported increased tibial loading after a 20 min run but no further increase after another 20‐min run starting 2 minutes after completion of the first. These different results are despite the fact the running speed was similar to the recreational group in the present study. It is unclear why the discrepancies occur, but it could be the result of the intensities of the respective protocols. In the previous study, the participants' Rating of Perceived Exertion score was 10–12 out of 20 (Rice et al., 2019), compared with approximately 17 in the present study (Sanno et al., 2018). Khassetarash et al. found no differences in strained volume of the tibia after 30 min of downhill running (Khassetarash et al., 2023), but it is not clear if this finding is specific to the downhill nature of the running. Strained volume may be a better predictor of bone failure than peak magnitude (Haider et al., 2021), and investigation of strained volume using participant specific imaging and density data would aid understanding of the present findings.

The finding of reduced peak loading and the reduced rate of damage accumulation throughout the run is indicative of a change in running gait that may be driven by fatigue of given muscles or muscle groups. From this same dataset, it was previously observed that there was a shift of positive work done at the lower limb joints from distal to proximal, that is, reduced work done at the ankle joint (Sanno et al., 2018). This shift of work done could be the result of ankle plantar flexor fatigue due to the relatively high demand placed on these groups of muscles during running. It has been suggested that muscles play a role in minimizing the external loading applied to the tibia during running by contributing to bending in the opposite direction to the reaction forces (Beck, 1998), and therefore, this ability is impaired when fatigued. However, according to the results presented here, that is not the case. The direction of tibial bending throughout stance is predominantly in the direction of triceps surae muscle contraction (Derrick et al., 2016; Meardon et al., 2014, 2015; Rice et al., 2019; Yang et al., 2014). Therefore, reduced activity of the triceps surae muscle group as a result of fatigue may explain the reduction in the magnitude of tibial bending observed in the present study. From a purely mechanistic perspective, this would suggest that reduced activity of the triceps surae muscle group may reduce tibial loading. Muscular activity was not monitored in this study, but previous research has shown reduced triceps surae muscle activity following a prolonged run (Ackermans et al., 2016; Girard et al., 2012; Saldanha et al., 2008; Weist et al., 2004). In summary, the results of the present study suggest that stress injury development from a mechanical perspective is not influenced by the reduction in work done at the ankle that occurs as a result of prolonged running. However, while it could be tempting to suggest that such reductions in triceps surae muscle activity and internal loading may offer some protection against tibial stress injury development, the cumulative damage increases with increasing distance completed to a much greater extent than any offsets that result from reduced peak loading per step. Thus, any protective effect is likely negligible.

While the aim of this study was to contribute to understanding of the risk of stress injuries, the findings should not be overinterpreted. The bones of competitive runners may be better adapted to withstand the loading experienced during running, as a result of their greater exposure to running. Therefore, injury risk cannot be directly compared between the running groups as the loading and remodeling capacity of each group is unknown. Additionally, the differences in tibial loading observed here may not hold across all speed and step frequency combinations. It is necessary to quantify this across a variety of speeds and step frequency manipulations, as well as in female runners, before generalizing. Furthermore, in addition to greater tibial loading experienced by competitive runners during one 10‐km run, training volume and recovery time are likely important contributors to stress injury development, and competitive runners likely have a higher training volume than recreational runners.

Step length increased throughout the run by only 1.8% in the present study, reflecting an average increase of 0.01 normalized lengths. This occurred alongside a nonsignificant (*p* = 0.062) decrease in step frequency of 0.05 Hz which may explain how an increase in stride length was possible at a fixed running speed, despite no apparent decrease in step frequency. However, these changes can be considered negligible and not practically meaningful.

The most significant limitation of this work is the lack of evidence relating these modeling approaches to injury outcomes, or other clinical indicators. In particular, the cumulative damage variable was derived from the peak internal moment which is a surrogate for bone loading and does not account for the material properties or specific geometry of the bone. As such, these findings can aid understanding and discussion of stress injury development, but recommendations to inform training in the context of injury risk should be made cautiously.

Modeling approaches rely on assumptions that introduce limitations. One of the major challenges of this type of modeling is the estimation of muscular forces using static optimization. This relies on the accuracy of the joint moments obtained, which can be influenced by both the data collection procedures and the hardware. Additionally, individual muscular strategies that may occur as a result of genetics or training adaptations will not be captured. Furthermore, the muscle positions relative to the tibia are not participant‐specific and so will not accurately represent differences between individual participants. The present study focused only on medial–lateral bending moments, which are the greatest in magnitude and are not influenced by the choice of joint moment constraint during static optimization (Baggaley et al., 2023), but it is important to note that the cumulative influence of all three components may be relevant to the progression of overuse injuries of the tibia. In addition, only one specific cross‐section of the tibia has been modeled here, and thus is not representative of the entire tibia.

Another limitation is that all the participants in the present study were male. Females should be included in studies wherever it is relevant, as is the case here, particularly because females experience a higher incidence of stress fractures than males (Wentz et al., 2011). Females of a high competitive level run more slowly than males of an equivalent level, and it is unclear how their results would compare to competitive males and recreational males. The running protocol in the present study is somewhat realistic of that which might be undertaken by both recreational runners and competitive runners in any given training session. However, this represents a single mode of running training, and different results may be observed when running overground, outdoors, at a non‐fixed running speed, and over different distances.

This study focused on the potential for damage accumulation as a result of running, but it is important to remember that loading of bones can also result in beneficial remodeling when the loading is not excessive and/or recovery time is sufficient. In addition, factors other than mechanics influence bone's risk of stress injury, such as nutritional and hormonal status, and those factors were not considered here.

## 5 CONCLUSIONS

Peak tibial loading and the rate of damage accumulation decrease throughout a demanding 10‐km run, and this suggests that running‐induced changes in running mechanics may not be a predominant mechanism for stress injury development. Additionally, competitive runners who complete a 10‐km run at a faster running speed than recreational runners generate considerably higher peak tibial loading, with indicators of greater cumulative tibial loading, despite requiring fewer steps of shorter duration to complete the run.

## CONFLICT OF INTEREST STATEMENT

Nothing to declare.

## Data Availability

The data that support the findings of this study are available from the corresponding author upon reasonable request.
